# A Sprayable and Visible Light Rapid-Cured Strippable Film for Surface Radioactive Decontamination

**DOI:** 10.3390/polym14051008

**Published:** 2022-03-02

**Authors:** Huiyuan Zhang, Hongxing Zhang, Wenchao Zhu, Hailing Xi, Bomou Ma, Yong He

**Affiliations:** 1 State Key Laboratory of NBC Protection for Civilian, Beijing 102205, China; 2190485@mail.dhu.edu.cn (H.Z.); zhx78668@126.com (H.Z.); 15811043200@139.com (W.Z.); 2 State Key Laboratory for Modification of Chemical Fibers and Polymer Material, College of Materials Science and Engineering, Donghua University, Shanghai 201620, China

**Keywords:** visible light curing, strippable film, radioactive decontamination

## Abstract

Strippable film is effective for removing radioactive contamination. However, it still has some limitations, such as the long curing time (about 30 min~24 h) and the requirement of organic solvents. To address these issues, we report a simple protocol to prepare strippable decontamination films using liquid polybutadiene (LPB) and tert-butyl acrylate (TBA) as the raw materials without solvent and using camphorquinone/ethyl 4-dimethylaminobenzoate (CQ/EDB) as a photoinitiator, where the film was formed under household LED panel light or daylight irradiation for about 540 s. After a thorough study of viscosity, real-time Fourier transform infrared (RT-FTIR spectra), gel and volatile organic compound (VOC) contents, mechanical properties and decontamination efficiency, the optimum composition and curing conditions were determined for the decontamination strippable film. VOC content is as low as 12.7 ± 0.7% and the resultant strippable film exhibits good mechanical performances with a tensile strength of up to 5.4 ± 0.4 MPa and elongation of up to 66.6 ± 13%. Most important, the decontamination efficiencies of this strippable film for ^133^CsCl on glass, ceramic and metal surfaces reach up to 98.1%, 94.3% and 97.6%, respectively.

## 1. Introduction

Nuclear energy, a zero-emission clean energy source, has been proposed as an alternative to fossil fuels because it does not emit huge amounts of pollutants such as carbon dioxide, nitrogen and sulfur dioxide into the atmosphere [1]. However, the major problem of nuclear energy is the potential of accidents and the large amounts of radioactive materials in nuclear power plants polluting the environment and endangering human life. 

Nowadays, various methods have been reported to remove radioactive decontamination, such as high-pressure water washing [2,3], mechanical grinding [4], ultrasound [5], electrochemical methods [6], gel [7,8] and foam [9]. Their application is limited by costs, the type of contamination, secondary pollutants and other factors. Strippable films display many advantages due to their low cost, easy application, low waste generation after decontamination, and compatibility with different surfaces (metal, glass, ceramic, cement, etc.) [10].

Currently, the commercialized strippable films used for decontamination and decommissioning are ALARA 1146, DeconGel 1108, TLC Free, Isotron Radblock and InstaCote CC Wet/CC Strip. ALARA 1146 is a water-based strippable film with a solids content of 41% and a film-forming time of about 18 h. It achieves the decontamination factors of 30–100% depending on the substrate [11]. DeconGel is a high-viscosity hydrogel and forms a strippable film in 12 h, it reaches the decontamination factors of up to 82–100% for hard nonporous surfaces (aluminum, copper, carbon steel, stainless steel, glass, ceramic, etc.) [12,13]. TLC Free, a water-based strippable film, can be used for nuclear reactor cavity decontamination, and 30 m^2^ of coated surface generates approximately 0.03 m^3^ of noncompacted radioactive waste [14]. In addition, Isotron corporation developed a rubber-based strippable film—Isotron Radblock. In 2008, the U.S. Environmental Protection Agency (EPA) thoroughly investigated the decontamination performances of TLC Free and Isotron Radblock. These two kinds of films were sprayed onto concrete contaminated with ^137^Cs and then cured overnight and removed from the concrete surface. The results show that Isotron Radblock reaches decontamination efficacy of 76.2 ± 7.4%, while TLC Free only reaches 32.0 ± 9.9% [15,16]. That is because Isotron Radblock has chemical chelation and physical bonding with ^137^Cs, while TLC Free only has physical bonding with ^137^Cs. InstaCote CC Wet and CC Strip are mainly composed of polyurea elastomers, which are formed by the mixed reaction of isocyanate-terminated compounds with amine compounds containing at least two reactive amine groups. CC Wet is the first step in controlling contamination. CC Strip is used to firmly adhere to CC Wet to form a dense sac-like film. They can be completely stripped from the surface together. This kind of strippable film has a short drying time (less than 1 h) and a high decontamination factor (CC Strip in plutonium glovebox reaches decontamination factor of 100%) [14]. 

Although these commercial formulations are successful, it is still necessary to perform further research to improve their performances. Gray et al. [17] developed a polymeric smart coating that is capable of both showing the contaminated areas by color change and removing contaminants from surfaces. Liu et al. [18] prepared self-brittle strippable film with poly(methyl methacrylate)-*block*-poly(methacrylate diblock copolymer) (PMMA-b-PMAA) as the main component and ethanol as the solvent. This self-brittle strippable film could split into fragments and be collected or cleaned up with automatic machines, avoiding human contact with radioactivity. Zhang et al. [19] prepared a low-temperature film-forming strippable film that takes acrylate copolymer as the main body and ethyl acetate as the solvent. The drying time of the film is about 14 h. 

In addition to acrylic copolymer, polyvinyl alcohol (PVA) is often chosen as the main component in decontamination formulations, and the modification of PVA water-based strippable film is also a research hotspot [20]. For example, Toader et al. [10] modified PVA water-based strippable film with EDTA and bentonite as a chelating agent and successfully applied PVA strippable film to heavy metal decontamination, and the decontaminating rate for Hg^2+^ on a copper sheet reached 75.9%. In addition, Banerjee [21] and Luong et al. [22] employed glycerin as the plasticizer and acetic acid or citric acid as the chelating agent to modify PVA strippable film. After drying for 24 h, this strippable film can remove ^137^Cs and ^84^Sr with a decontamination rate of more than 90%. Yang et al. [7,8,23,24,25] from the Korea Atomic Energy Research Institute developed a series of PVA-based hydrogels containing ^137^Cs adsorbent. Interestingly, these hydrogels have reversible crosslinking characteristics. Moreover, the adsorbent can be separated from the hydrogel to reduce the volume of radioactive waste. However, the hydrogels cannot be stripped and can only be removed by washing with water.

In the field of radioactive decontamination, strippable films have been explored for a long time, but most previously reported strippable films require the use of large quantities of solvents such as ethanol and ethyl acetate. They have obvious problems of volatile organic compound (VOC) emissions which are harmful to the environment and human safety and greatly limit their application in large-scale decontamination. Water-based strippable film is a new hotspot due to the low VOC emissions and high utilization rate of raw materials, but overcoming the long drying time remains a challenge.

Recently, ultraviolet (UV) curable strippable film has attracted a great deal of attention because of its significant advantages such as less environmental pollution, shorter drying time and higher efficiency in production [26,27]. Sun et al. [28] employed poly(diallyl phthalate) (P-DAP) as the solid component, tri(propylene glycol) diacrylate (TPGDA) as the active diluent and bis(2,4,6-trimethylbenzoyl) phenylphosphorous oxide (BAPO) as the photoinitiator to fabricate a UV-curable strippable film upon mercury lamp irradiation for 30 s; this film exhibited an excellent protective effect and was easy to peel off. Tao et al. [29] reacted tris(4-allyl-2-methoxyphenyl) phosphate (TAMPP) with a multifunctional thiol to prepare a flexible and transparent film via thiol−ene “click” photopolymerization with mercury lamp (365 nm) irradiation for 5 min. Shen et al. [30] developed a photocurable temporary protective film with modified epoxy acrylate resin and Photoinitiator 1173. After 2 min irradiation under a UV lamp, the cured film could resist acid and organic solvents. Nevertheless, it could be completely removed only when exposed to alkali solution, which damages the substrate. In general, the trifunctional allyl compound or resin in the above three UV-cured strippable film formulations resulted in their high viscosity and greatly limited their application in large-scale spraying. Meanwhile, these systems require UV light to be activated, but UV light is harmful to eyes. So far, there are few reports of achieving rapid-cured strippable film under visible light. The use of low-intensity visible light would be a significant improvement because visible light could avoid using expensive photochemical equipment, and it is safer and less energetic compared with UV light.

**Scheme 1:**
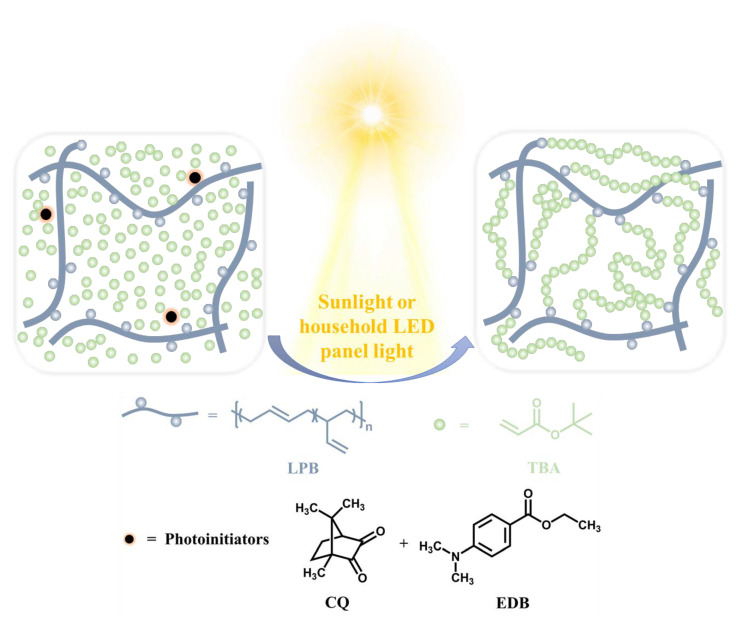
The reaction scheme of the strippable film.

As stated above, the development of solvent-free, short-curing-time, low-viscosity and visible-light-initiated strippable films is meaningful for practical use. Here, we present a simple strategy to prepare sprayable and strippable films based on commercially available chemicals (Scheme 1). We used camphorquinone/ethyl 4-dimethylaminobenzoate (CQ/EDB) as a photoinitiating system and liquid polybutadiene (LPB) and tert-butyl acrylate (TBA) as monomers to obtain strippable films exhibiting rubber elasticity without the solvent. Strippable films could be rapidly cured in 540 s under irradiation with visible light, such as household LED panel light and sunlight. The resultant strippable films possess good mechanical properties, high decontamination efficiency and excellent strippability. In addition, the use of commercially available raw materials and the simplicity of this strippable film make it suitable for industrial adoption.

## 2. Experiment

### 2.1. Materials

Liquid polybutadiene (LPB, Lithene Active 1000, M_n_ = 3000 gꞏmol^−1^, 1,2 addition ratio = 70%) was obtained from Synthomer (Shanghai, China). Tert-butyl acrylate (TBA, 99%, inhibited with 4-methoxyphenol) and camphorquinone (CQ, >98%) were purchased from Shanghai Aladdin Bio-Chem Technology Co., Ltd (Shanghai, China). Ethyl 4-dimethylaminobenzoate (EDB, 97%) was from Shanghai Bidepharm Bio-Chem Technology Co., Ltd. (Shanghai, China). They were all used as received. The chemical structures of these chemicals are shown in Scheme 1. 

### 2.2. Sample Preparation

#### 2.2.1. Preparation of TBA Homopolymer (PTBA) by Photopolymerization

A series of TBA homopolymers (PTBA) were prepared under household LED panel light irradiation (light intensity: 50 mW/cm^2^, wavelength: 410–760 nm) using CQ/EDB as the photoinitiating system. The loading of CQ/EDB was controlled to be 0.2 phr/0.2 phr, 0.5 phr/0.5 phr or 1 phr/1 phr while the irradiation time was set to be 200, 400 or 600 s. After the photopolymerization, PTBA was purified by precipitation from its dichloromethane solution in pentane.

#### 2.2.2. Preparation of LPB/TBA Solutions 

LPB and TBA were stirred together for 30 min to ensure thorough mixing, and then the CQ/EDB photoinitiating systems were added into the mixture and stirred in the dark for another 10 min to obtain homogeneous solutions. 

#### 2.2.3. Preparation of Strippable Film via Visible Light Curing

The resulting LPB/TBA solutions were sprayed in the PTFE molds and irradiated by a household LED bulb or sunlight to prepare strippable films. The average thickness of the films was 1 mm. The light intensity of the household LED bulb on the surface of films was controlled at about 50 mW/cm². In the experiment, LPB/TBA solutions with the initiators were wrapped in aluminum foil to avoid the influence of light as much as possible.

### 2.3. Structural Characterization

Gel permeation chromatography (GPC): The molecular weights of PTBA were determined at 35 °C on an size exclusion chromatography system (1260 Infinity II, Agilent, America). Hexafluoroisopropanol (HFIP) was used as the eluent at a flow rate of 0.3 mLꞏmin^−1^, narrowly dispersed polymethyl methacrylate (PMMA) was used as standards and Agilent PL HFIPgel column (4.6 × 250 mm, 9 μm) was used as the chromatographic column.

Viscosity: The viscosity of the resultant LPB/TBA solution was measured at 30 °C on a rheometer (RS 150L, HAAKE, Germany) with a gap of 0.052 mm.

Real-time Fourier transform infrared spectroscopy: Real-time Fourier transform infrared spectroscopy (NEXUS-670, Nicolet, America) was utilized to quantitatively characterize the photopolymerization kinetics of the LPB/TBA solution. One drop of the LPB/TBA solution was deposited between two CaF_2_ windows to reduce the oxygen inhibition effect, where the thickness of the LPB/TBA solution was controlled to be about 25 μm [31]. The CaF_2_ window was then inserted into the sample holder and exposed to household LED panel light with an intensity of 50 mW/cm² to trigger the photopolymerization of TBA and LPB. At the same time, the FTIR scanning was started, and the spectra were recorded per 60 s. As the double bond of 1,4-addition LPB remains unchanged during the polymerization, its absorption band at 908 cm^−1^ was used as an internal reference. The double bond conversions (DBCs) of TBA and LPB were determined according to Formulas (1) and (2) [32,33,34], respectively:

(1) $$\mathrm{DBC}\;\mathrm{of}\;\mathrm{TBA}=\left(1-\frac{{\left(\frac{A_{848}}{A_{908}}\right)}_{t}}{{\left(\frac{A_{848}}{A_{908}}\right)}_{0}}\right)\times 100\%$$

(2) $$\mathrm{DBC}\;\mathrm{of}\;\mathrm{LPB}=\left(1-\frac{{\left(\frac{A_{993}}{A_{908}}\right)}_{t}}{{\left(\frac{A_{993}}{A_{908}}\right)}_{0}}\right)\times 100\%$$

where *A*_993_*, A*_908_ and *A*_848_ are the areas of C=C bending vibration (γ _= C-H_) of 1,2-addition LPB at 993 cm^−1^, 1,4-addition LPB at 908 cm^−1^ and TBA at 848 cm^−1^, respectively. *t* is the irradiation time, and 0 means the irradiation time of 0 s. The measurements were performed three times for each sample and the average was recorded as the result.

Gel content: The gel content of strippable films was determined by soaking the strippable film sample (original weight, *W*_0_) in CHCl_3_ for 48 h with the CHCl_3_ refreshed each 24 h. After that, the insoluble strippable film was placed in a vacuum and dried at 80 °C to a constant weight (*W*_1_). The gel content was calculated from the Formula (3):

(3) $$\mathrm{Gel}\;\mathrm{content}=\frac{W_{1}}{W_{0}}\times 100\%$$

The measurements were performed three times for each sample and the average was recorded as the result. 

Volatile organic compound (VOC) content: The VOC content of strippable films was determined from the mass loss of the sample during the drying under vacuum at 80 °C for 6 h as given in Formula (4):

(4) $$\mathrm{VOC}=\frac{M_{0}-M_{1}}{M_{0}}\times 100\%$$

where *M*_0_ is the initial weight of the films and *M*_1_ is the weight after drying. The measurements were repeated three times and the average value was reported.

Tensile tests: Tensile tests were performed to characterize mechanical properties of strippable films by using an MTS E42 tensile-testing machine with a stretch rate of 20 mmꞏmin^−1^. The strippable film samples were prepared by placing LPB/TBA solution in a PTEF mold (60 ×20 ×1 mm) upon irradiation of visible light. At least six specimens were tested and averaged for each sample.

Decontamination efficiency (DE): Due to the limitation of experimental conditions, we used cesium chloride (^133^CsCl) to simulate ^137^Cs radioisotopes. Firstly, about 0.3 g CsCl (*C*_0_) was sprayed on a glass, ceramic or metal surface, and the contaminated diameter was controlled at about 30 mm. Subsequently, LPB/TBA solution (LPB content: 25 wt%, CQ/EDB loading: 0.5 phr/0.5 phr) was poured onto the contaminated surface and immediately irradiated for 540 s with household LED panel light. The resultant film was stripped and the mass of the remaining CsCl (*C*_1_) was measured. Finally, the decontamination efficiency (DE) was calculated according to Formula (5):

(5) $$\mathrm{DE}=\frac{C_{0}-C_{1}}{C_{0}}\times 100\%$$

where *C*_0_ is the initial mass of the CsCl and *C*_1_ is the remaining mass of the CsCl on the contaminated surface after decontamination. The measurements were repeated three times and the average value was reported.

## 3. Results and Discussion

### 3.1. Molecular Weight of PTBA

Before studying the visible light curing behavior of the LPB/TBA solution, it was necessary to investigate the photopolymerization of TBA. GPC was employed to analyze the molecular weight of PTBA. 

**Figure 1:**
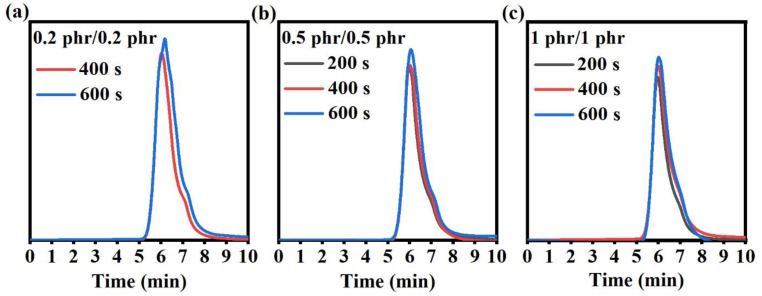
GPC spectra of PTBA prepared with different loadings of CQ/EDB: (**a**) 0.2 phr/0.2 phr; (**b**) 0.5 phr/0.5 phr; (**c**) 1 phr/1 phr, under the household LED panel light irradiation for different times.

The molecular weights of PTBA prepared with different loading of CQ/EDB under the household LED panel light irradiation for different times are shown in Table 1 and Figure 1. With the increase in irradiation time, the weight-averaged molecular weight (M_w_) and the number-averaged molecular weight (M_n_) declined. After a certain irradiation time, the decreased molecular weights of PTBA result from the increased initiating species during the irradiation. Interestingly, when the loading of photoinitiating system and the irradiation time were 0.2 phr/0.2 phr and 400 s, the resultant strippable films possessed high molecular weights (M_n_ = 184 kgꞏmol^−1^, M_w_ = 335 kgꞏmol^−1^), allowing the development of strippable films with good mechanical properties. 

### 3.2. Viscosity of the LPB/TBA Solution

**Figure 2:**
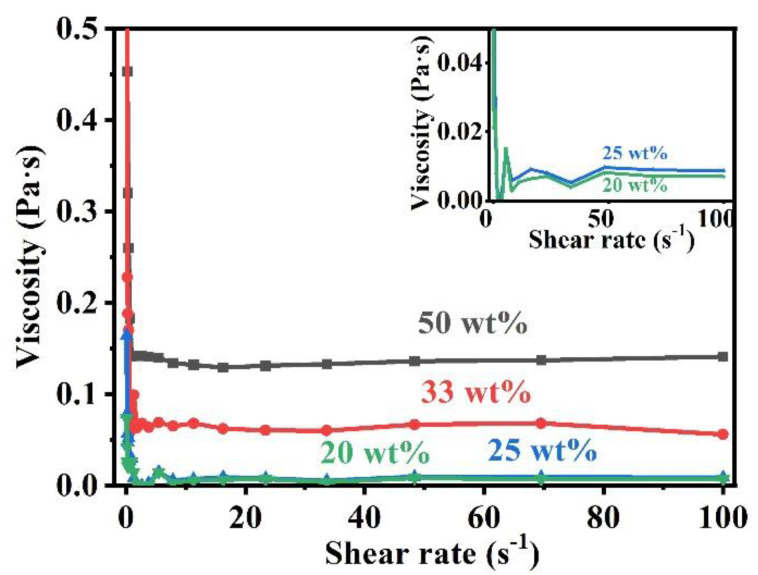
The viscosity of LPB/TBA solution with different liquid polybutadiene (LPB) contents.

The viscosity of the LPB/TBA solution is an important parameter affecting the application method. Low-viscosity solutions are conducive to spraying and the improved molecular mobility contributes to the solution wetting the substrate surface. Furthermore, the decontamination efficiency of the strippable film is improved [22]. Therefore, the effect of LPB content on the viscosity of LPB/TBA solutions was investigated, as shown in Figure 2. The figure shows that the LPB/TBA solutions display a shear-thinning character, the viscosity decreases quickly with the shear rate and remains the same after 1 s^−1^. The viscosity is closely associated with the content of LPB and increases with the LPB content. However, when the content of LPB is high, up to 50 wt%, the viscosity of LPB/TBA solution is only 0.15 Paꞏs.

### 3.3. Polymerization Mechanism of the Strippable Films

The strippable films were synthesized by visible light polymerization, as illustrated in Scheme 1. Upon exposure to visible light, CQ is converted to an excited state before forming free radicals (CQ−H•) by abstracting a hydrogen atom from EDB [35]. TBA and side chains of 1,2-addition LPB are triggered by CQ−H• free radicals. TBA is rapidly polymerized into linear polymers (PTBA); at the same time, the double bonds in side chains of LPB are polymerized with the double bonds in PTBA to form a cross-connected network. 

### 3.4. Double Bond Conversion (DBC) and Photopolymerization Rate of TBA and LPB

In the real-time Fourier transform infrared (RT-FTIR) spectroscopy experiments, household LED panel light (50 mW/cm^2^) was used to explore the influence of photoinitiators on double bond conversions (DBCs) and photopolymerization rates of TBA and LPB.

**Figure 3:**
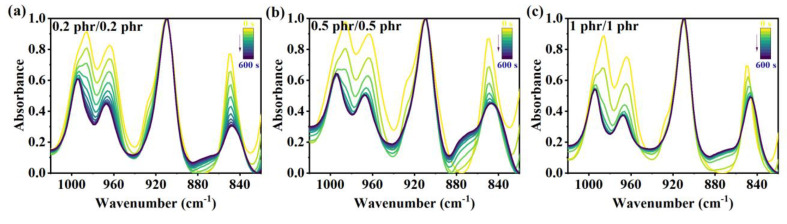
The FTIR spectra ranging from 1020 to 820 cm^−1^ for liquid polybutadiene/tert-butyl acrylate (LPB/TBA) solution during irradiation (household LED panel light, 50 mW/cm^2^) in the presence of different loadings of CQ/EDB photoinitiating systems: (**a**) 0.2 phr/0.2 phr; (**b**) 0.5 phr/0.5 phr; (**c**) 1 phr/1 phr, the spectra recorded per 60 s.

The FTIR spectra of LPB/TBA solution in the presence of different loadings of CQ/EDB systems were recorded and compared every 60 s during the photopolymerization process (Figure 3). Strong bands in the ranges of 1020~979, 941~887 and 887~825 cm^−1^ reflect the C=C bending vibration (γ = _C-H_) of 1,2-addition LPB, 1,4-addition LPB and TBA, respectively [36,37]. As the double bond of 1,4-addition LPB remains unchanged during the polymerization, the corresponding absorption band is used as an internal reference. Before the household LED panel light irradiation, strong C=C bending vibration bands of 1,2-addition LPB and TBA were observed. After the irradiation for 360, 240 and 180 s, the maximum intensity of these two bands decreases, in the presence of 0.2 phr/0.2 phr, 0.5 phr/0.5 phr and 1 phr/1 phr loadings of CQ/EDB photoinitiating systems, respectively. This demonstrated that the photopolymerization of 1,2-addition LPB and TBA occurred at the same time and the high loading of photoinitiating systems resulted in a faster photopolymerization rate. As LPB and TBA form a transparent solution before irradiation, simultaneously photopolymerize with the irradiation and form a transparent film after irradiation, it is reasonable to conclude that copolymerization of LPB and TBA occurred and the strippable film was successfully synthesized.

**Figure 4:**
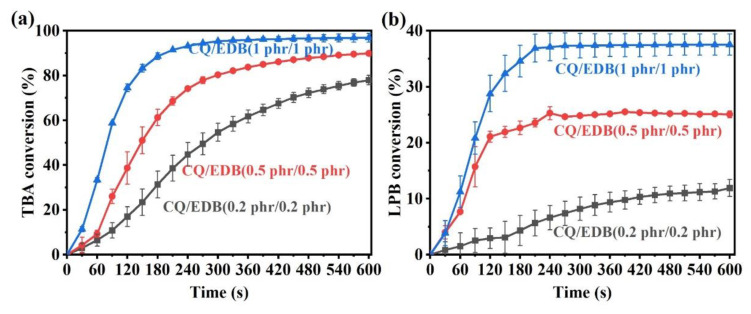
The double bond conversion (DBC) of (**a**) TBA and (**b**) LPB under household LED panel light (50 mW/cm^2^) irradiation for different times.

The areas of these two bands were calculated, and the double bond conversions (DBCs) of TBA and LPB were determined according to Formulas (1) and (2), respectively. The results are depicted in Figure 4. With the increase in irradiation time from 0 to 600 s, the DBC of TBA increased linearly in the first 180 s and then increased slowly and finally leveled off (Figure 4a). Similarly, at the first 120 s, the DBC of LPB increased linearly, and then it leveled off gradually (Figure 4b). The loading of CQ/EDB photoinitiating systems greatly influenced the photopolymerization rate and DBC of monomers (TBA and LPB). The average photopolymerization rate and final DBC of TBA and LPB are summarized in Table 2. Both values increased with the loading of CQ/EDB photoinitiating systems. However, in the presence of the highest loading of CQ/EDB photoinitiation systems, the DBC of LPB was only 37.5 ± 1.9%, while the DBC of TBA was high, reaching up to 96.8 ± 1.9%, which may be due to the double bonds in 1,2-addition LPB side chains being inactive.

### 3.5. Gel Content

**Figure 5:**
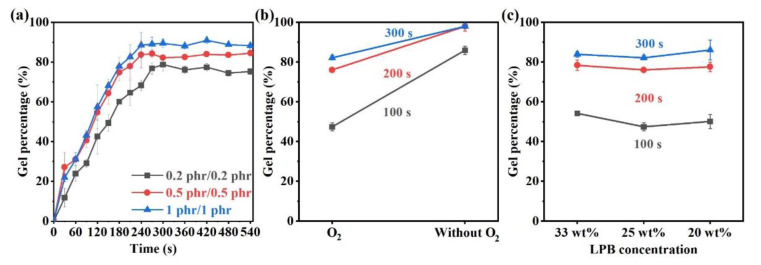
Gel content of strippable films (LPB content: 25 wt%, prepared with O_2_) with different loadings of CQ/EDB for different irradiation times (**a**); the gel content of strippable films (LPB content: 25 wt%, CQ/EDB loading: 0.5 phr/0.5 phr), prepared with O_2_ or without O_2_ (**b**); the gel content of strippable films (CQ/EDB loading: 0.5 phr/0.5 phr, prepared with O_2_) with different contents of LPB (**c**), under household LED panel light irradiation.

In order to roughly evaluate the crosslinking degree of strippable films in the photopolymerization reaction, the gel content was determined every 60 s, and the results are shown in Figure 5a. The figure shows that the gel content increased with the photopolymerization time and leveled off finally, indicating that crosslinking degree was closely related to irradiation time at the beginning of polymerization. Besides, the crosslinking degree was also closely associated with the loading of photoinitiating systems. Anyway, the strippable films with the low loading of CQ/EDB had a low crosslinking degree due to the low polymerization rate, which is consistent with the results obtained from the polymerization kinetics (Section 3.4) [32]. In addition, as the loading of photoinitiators decreased from 1 phr/1 phr to 0.2 phr/0.2 phr, the crosslinking degree decreased from 88.2 ± 1.1% to 75.3 ± 1.4%. This is because the double bonds in 1,2-addition LPB side chains are difficult to polymerize and the photoinitiator cannot fully initiate them, which leads to the low double bond conversion of LPB in Figure 4b. Therefore, the decreased crosslinking degree of the strippable films with low photoinitiator loading results from inactive double bonds in LPB side chains.

To study the effects of oxygen inhibition on crosslinking degree of strippable films, the gel content of the strippable films under aerobic and anaerobic conditions was investigated at different polymerization times; the results are shown in Figure 5b. It can be seen that gel content of strippable films without O_2_ increased from 47.5 ± 2.0% to 85.9 ± 2.1% under 100 s irradiation and from 82.2 ± 1.1% to 98.1 ± 0.5% under 300 s irradiation. These results illustrate that O_2_ hindered the polymerization of monomers on the surface of the film, thus reducing the crosslinking degree of the film.

Figure 5c depicts the influence of LPB content on the crosslinking degree. At the same polymerization time, the content of LPB had no obvious effect on the gel content, reflecting that the crosslinking degree was independent of the monomer ratio of strippable films. 

### 3.6. VOC Content

**Figure 6:**
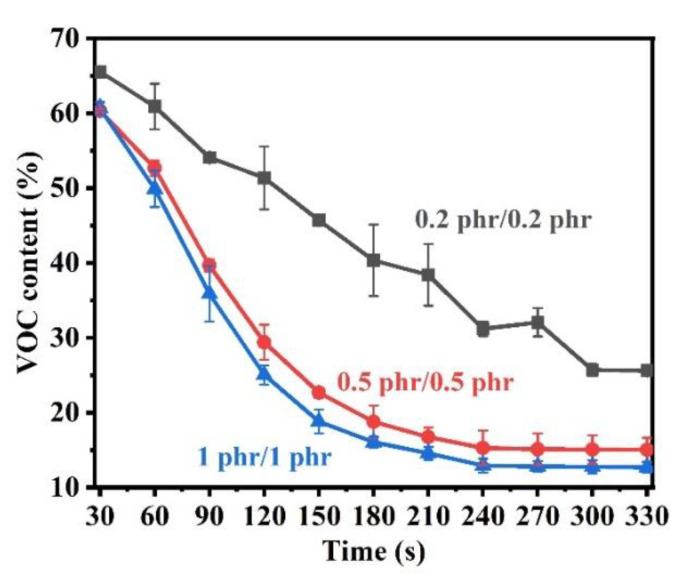
The VOC content of strippable films with different loadings of CQ/EDB for different irradiation times (LPB content: 25 wt%, prepared with O_2_), under household LED panel light irradiation.

Most strippable films use organic solvents as dispersants, resulting in high VOC content. Because high VOC content poses a great threat to the environment and human health, VOC content is an important index for evaluating the safety of strippable film. VOC content of strippable film with the photopolymerization time is recorded in Figure 6. At 0.2 phr/0.2 phr CQ/EDB loading, the final VOC content of strippable films was 25.6 ± 0.6%, while it gradually decreased and stabilized at 15.0 ± 1.6% and 12.7 ± 0.7% at 0.5 phr/0.5 phr and 1 phr/1 phr CQ/EDB loading, respectively. The loading of CQ/EDB has a significant impact on the final VOC content of strippable films by affecting the ratio of uncrosslinked monomers in films (as shown in Figure 5a).

It is worth noting that monomers on the surface of the strippable film are not easily polymerized, leading to the high VOC content, because of the effect of oxygen inhibition on strippable film (as shown in Figure 5b). However, compared with the high VOC content of solvent-based strippable film (the solvent-based strippable films generally contained volatile organic components (VOCs) of 29–59 wt% [14]), our strippable film has certain advantages.

### 3.7. Mechanical Properties

The mechanical properties of the films at different conditions were also examined to explore their application potentials as strippable films because the strippable films used for decontamination must be resistant and elastic. 

**Figure 7:**
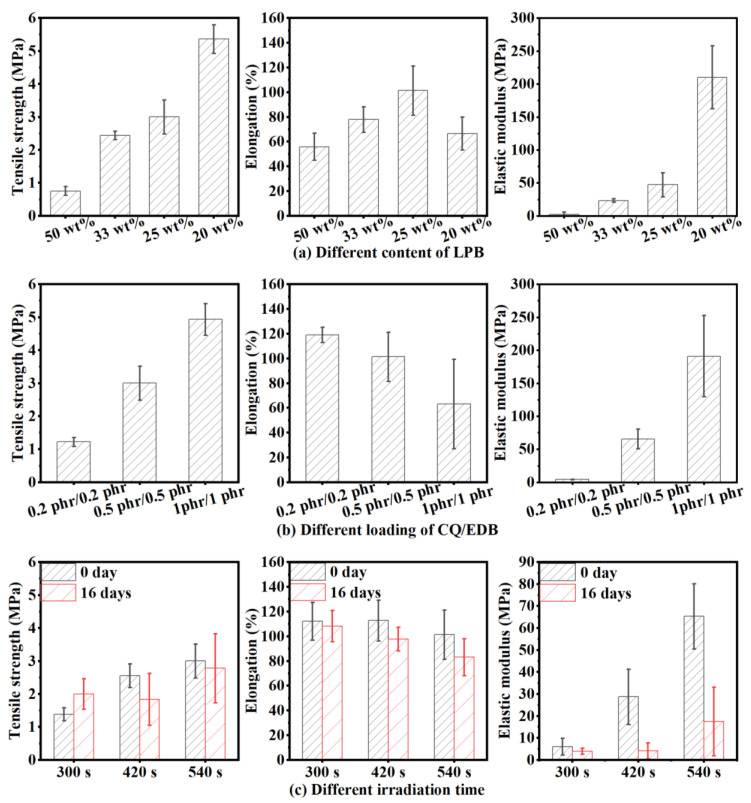
Tensile strengths, elongations at break and elastic moduli for strippable films (**a**) with different content of LPB (CQ/EDB loading: 0.5 phr/0.5 phr, prepared without O_2_, household LED panel light irradiation time: 540 s); (**b**) with different loadings of CQ/EDB (LPB content: 25 wt%, prepared without O_2_, household LED panel light irradiation time: 540 s); (**c**) under household LED panel light irradiation for different times (LPB content: 25 wt%, CQ/EDB loading: 0.5 phr/0.5 phr, prepared without O_2_), the black columns refer to LPB/TBA solution stored for 0 days and red ones refer to LPB/TBA solution stored for 16 days.

As shown in Figure 7a, the content of LPB greatly influenced the mechanical behaviors of strippable films. As LPB content decreased from 50 to 20 wt%, the tensile strengths and elastic moduli of films increased from 0.7 ± 0.1 MPa and 1.8 ± 0.4 MPa to 5.4 ± 0.4 MPa and 210 ± 36 MPa, while the elongations at break first increased and then decreased with maximum values of 101 ± 20% at LPB content of 25 wt%. The mechanical properties of strippable films are closely related to the molecular weight. However, M_n_ of LPB is only 3000 gꞏmol^−1^, whereas that of PTBA is as high as 18 × 10^4^ gꞏmol^−1^. Therefore, the addition of LPB leads to a decrease in tensile strengths and elastic moduli.

The effect of CQ/EDB photoinitiating systems on the mechanical properties of strippable films is shown in Figure 7b. The tensile strengths and elastic moduli of strippable films increased with the loading of CQ/EDB, whereas the elongations at break decreased with it. The tensile strengths achieved the maximum value of 4.9 ± 0.4 MPa at CQ/EDB loading of 1 phr/1 phr. Therefore, doping of CQ/EDB increased the density of crosslinking structure (as shown in Figure 5a), which contributes to the increased tensile strength and elastic moduli.

Owning to the photopolymerization process, the mechanical properties of the strippable films are dependent on the irradiation time of household LED panel light. As the irradiation time increased from 300 to 540 s, the tensile strengths and elastic moduli of the films increased from 1.4 ± 0.1 MPa and 6.1 ± 4 MPa to 3.0 ± 0.5 MPa and 65.4 ± 15 MPa, accompanying a compromise of elongations at break from 112 ± 15% to 101 ± 20% (Figure 7c). The irradiation time is associated with the molecular weights of polymer chains and the densities of physical crosslinks. In this sense, the irradiation time was kept as 540 s for the preparation of other strippable films. 

The effect of storing time for LPB/TBA solutions was also investigated, as shown in Figure 7c, to explore their storage stability. After the LPB/TBA solution was stored for 16 days, the mechanical properties of the strippable film declined, which may be related to the volatilization of TBA during storage.

**Figure 8:**
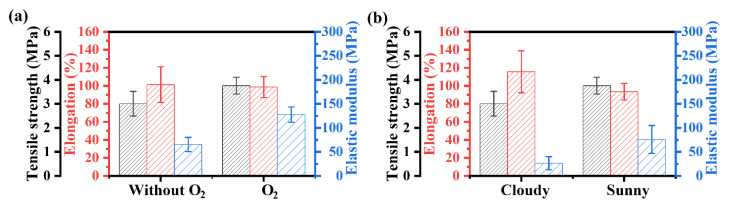
Tensile strengths, elongations at break and elastic moduli for strippable films (**a**) prepared without O_2_ or with O_2_ (LPB content: 25 wt%, CQ/EDB loading: 0.5 phr/0.5 phr, household LED panel light irradiation time: 540 s); (**b**) irradiated by sunlight on cloudy or sunny days for 540 s (LPB content: 25 wt%, CQ/EDB loading: 0.5 phr/0.5 phr, prepared without O_2_).

To study the effects of oxygen on the mechanical properties of strippable films, the mechanical properties of the strippable films prepared with O_2_ or without O_2_ were investigated, as shown in Figure 8a. Interestingly, the mechanical properties of the strippable films prepared under O_2_ increased. Therefore, under irradiation for sufficient time, the effect of O_2_ on mechanical properties can be ignored. 

Besides the household LED panel light irradiation, the strippable films can be formed under sunlight. Figure 8b shows the mechanical properties of strippable films irradiated by sunlight on cloudy or sunny days for 540 s. The sunlight intensity was about 0~50 mW/cm^2^ on cloudy days and 50~75 mW/cm^2^ on sunny days. On sunny days, tensile strengths and elastic moduli of the strippable film increased to 3.4 ± 0.2 MPa and 75.9 ± 29 MPa. The results suggest that the increase in sunlight intensity was conducive to improving the mechanical performances.

The temperature fluctuation during the irradiation may affect the crosslinking degree and thus cause errors in the mechanical properties of the strippable film. In addition, the fluctuation of O_2_ concentration during the irradiation and the change of the morphology of the film surface during the stripping procedure may cause errors in mechanical properties to a certain extent.

### 3.8. Decontamination Efficiency (DE)

**Figure 9:**
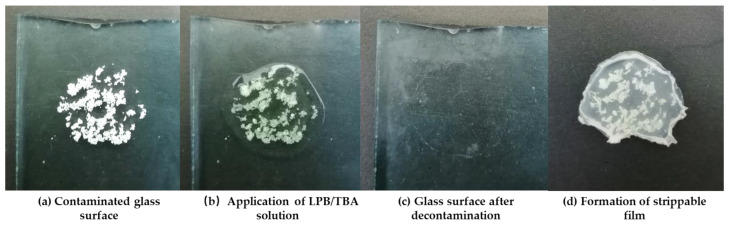
Decontamination procedure of strippable film on glass surface.

Decontamination efficiency is the most important property of the strippable film. The decontamination efficiency (DE) of the fabricated strippable film for hard nonporous surfaces is summarized in Table 3, and the decontamination process of this strippable film on a glass surface is shown in Figure 9. The DE reached 98.1%, 94.3% and 97.6% for removing cesium chloride (CsCl) from glass, ceramic and metal surfaces, respectively. From Figure 9, it can be seen that the strippable film has sufficient mechanical properties to ensure smooth stripping and thus remove almost all the pollutants on the surface. This demonstrates that the visible-light-curable films had excellent surface decontamination ability. Although the maximum capacity towards CsCl was not well investigated at this point, the present results suggested that the decontamination capacity of the film was not less than 0.48 g CsCl/g film under the studied conditions.

## 4. Conclusions

In this study, a novel sprayable and rapid-cured strippable film was developed in order to remove radioactive contamination from various types of surfaces. Under visible light (household LED panel light or sunlight) irradiation, the drying time of strippable film with LPB/TBA solution as the main component and CQ/EDB as the photoinitiator was shortened to 540 s. In addition, the viscosity of strippable film solution is as low as 0.15 Paꞏs, which is very suitable for spraying. Moreover, there is no solvent in the solution and the VOC content is as low as 12.7 ± 0.7%, avoiding environmental pollution. The results of the tensile tests show the tensile strength achieved the maximum value of 5.4 ± 0.4 MPa at the LPB content of 20 wt%, where elongation at break was 66.6 ± 13%. Most importantly, the decontamination efficiency (DE) of the film reached 98.1%, 94.3% and 97.6% for removing CsCl from glass, ceramic and metal surfaces, respectively.

## Figures and Tables

**Table 1:** Molecular weights of TBA homopolymers (PTBA) with different loadings of camphorquinone/ethyl 4-dimethylaminobenzoate (CQ/EDB) during different polymerization times.

Loading of CQ/EDB (phr/phr)	Time (s)	M_n_ (kgꞏmol^−1^)	M_w_ (kgꞏmol^−1^)	PDI
0.2/0.2	200	-	-	- *
0.2/0.2	400	184	335	1.82
0.2/0.2	600	155	303	1.96
0.5/0.5	200	185	350	1.89
0.5/0.5	400	177	334	1.89
0.5/0.5	600	171	324	1.89
1/1	200	185	353	1.91
1/1	400	163	336	2.07
1/1	600	148	318	2.16

* At 0.2 phr/0.2 phr CQ/EDB loading, no PTBA was formed after 200 s irradiation.

**Table 2:** Photopolymerization rate and final double bond conversion (DBC) of TBA and LPB with different loadings of CQ/EDB photoinitiating systems under household LED panel light irradiation.

Monomer	Loading (phr/phr)	Photopolymerization Rate * (s^−1^)	Final DBC (%)
TBA	0.2/0.2	0.18	77.9 ± 2.1
TBA	0.5/0.5	0.48	89.9 ± 1.0
TBA	1/1	0.84	96.8 ± 1.9
LPB	0.2/0.2	0.03	11.9 ± 1.5
LPB	0.5/0.5	0.26	25.0 ± 0.5
LPB	1/1	0.31	37.5 ± 1.9

* TBA: average photopolymerization rate for the first 180 s; LPB: average photopolymerization rate for the first 120 s.

**Table 3:** Decontamination efficiency of the strippable film on different surfaces.

Surface Type	DE (%)
Glass	98.1 ± 2.8
Ceramic	94.3 ± 5.7
Metal	97.6 ± 2.5
